# Distribution of the Harmful Bloom-Forming Cyanobacterium, *Microcystis aeruginosa*, in 88 Freshwater Environments across Japan

**DOI:** 10.1264/jsme2.ME19110

**Published:** 2020-02-20

**Authors:** Takafumi Kataoka, Kako Ohbayashi, Yuki Kobayashi, Hiroyuki Takasu, Shin-ichi Nakano, Ryuji Kondo, Yoshikuni Hodoki

**Affiliations:** 1 Faculty of Marine Science and Technology, Fukui Prefectural University, 1–1 Gakuen-cho Obama, Fukui, 917–0003, Japan; 2 Graduate School of Arts and Sciences, The University of Tokyo, 3–8–1 Komaba, Meguro, Tokyo 153–8902, Japan; 3 Faculty of Health Sciences Yamaguchi University Graduate School of Medicine, Minamikogushi 1–1–1, Ube, Yamaguchi 755–8505 Japan; 4 Faculty of Environmental Science, Nagasaki University, 1–14 Bunkyo-machi, Nagasaki, 852-8521, Japan; 5 Center for Ecological Research, Kyoto University, Otsu, Shiga, 520–2113, Japan

**Keywords:** *Microcystis aeruginosa*, 16S–23S ITS, qPCR, wide area distribution, genotype composition

## Abstract

*Microcystis aeruginosa* was quantitatively surveyed in 88 freshwater environments across Japan within 3‍ ‍weeks in 2011. In order to clarify the distribution pattern of *M. aeruginosa* at the intra-species level, three major genotypes, which were defined by 16S–23S rRNA inter-transcribed-spacer (ITS) regions, were selectively detected using quantitative real-time PCR assays. Of the 68 sites at which the *Microcystis* intergenic-spacer region of the phycocyanin (IGS-PC) gene was detected, the *M. aeruginosa* morphotype-related genotype (MG1) dominated in 41 sites, followed by the non-toxic *M. wesenbergii*-related genotype (MG3). A correlation analysis showed that total nitrogen and phosphate positively correlated with the abundance of IGS-PC, which positively correlated with microcystin synthetase gene abundance. A redundancy analysis of genotype compositions showed that pH positively correlated with the dominance of MG3 and negatively correlated with MG1, *i.e.*, both toxic and non-toxic genotypes. Our survey of *Microcystis* populations over a wide area revealed that MG1 is a dominant genotype in Japan.

Toxic cyanobacterial blooms are increasing public health and water quality management issues (Paerl, 2017). Massive blooms of *Microcystis*, one of the most pervasive bloom-forming cyanobacteria worldwide, with the exception of Antarctica (Zurawell *et al.*, 2005), cause serious damage to freshwater ecosystems and negatively impact the use of freshwater by human communities (Van Wichelen *et al.*, 2016). Due to human activities, global warming, and eutrophication, the frequency, intensity, and geographic distribution of harmful algal blooms have increased over recent decades (Paerl and Paul, 2012).

The genus *Microcystis* has been characterized based on its morphological features under a microscope, namely, cell size, colony morphology, and the appearance of cells embedded in a mucilage matrix (Komárek and Anagnostidis, 1986; 1999). The most common populations appear to be *M. aeruginosa*, *M. ichthyoblabe*, *M. novacekii*, *M. viridis*, and *M. wesenbergii* (Komárek, 1991). However, molecular studies reported a high level of similarity among these morphologically diverse *Microcystis* strains, which share more than 97% 16S rRNA gene sequence homology (Neilan *et al.*, 1997; Otsuka *et al.*, 1998), and more than 93% intergenic-spacer region sequence homology in the phycocyanin-coding (IGS-PC) gene (Neilan *et al.*, 1995; Bittencourt-Oliveira *et al.*, 2001). Therefore, *Microcystis* strains have been unified under the species *M. aeruginosa* based on a bacteriological taxonomic criterion, namely, DNA-DNA hybridization of >70% (Kondo *et al.*, 2000; Otsuka *et al.*, 2001).

Molecular analyses have been applied to detect, quantify, and evaluate the diversity and/or abundance of *Microcystis* communities in natural environments. Intra-species genotyping has been performed for several genes, such as the 16S rRNA gene (Yoshida *et al.*, 2005), IGS-PC (Neilan *et al.*, 1995), and *Microcystis* 16S–23S rRNA internal-transcribed-spacer (16S–23S ITS) region sequences (Yoshida *et al.*, 2008), and several genes have sufficient resolution to identify robust intra-specific genotypes. The quantification of gene copy numbers using real-time PCR (qPCR) is another genotyping method used in ecological studies. We developed qPCR methods to quantify intra-species genotypes based on variations in the 16S–23S ITS sequence by referencing gene sequences derived from specimens worldwide (Kataoka *et al.*, 2013). Furthermore, the gene cluster in microcystin synthetase (*mcy*) has been used to detect toxin-producing and potentially toxic cyanobacteria genotypes (Baker *et al.*, 2002).

Environmental studies demonstrated that *M. aeruginosa* is highly diverse at the intra-species level, not only among geographically distant ecosystems, but also during local bloom proliferation. Periodic studies of a local ecosystem showed temporal succession in the abundance and genotype variation of *Microcystis* (Yoshida *et al.*, 2005; 2007). In contrast to temporal studies, few studies have focused on spatial distributions within the *Microcystis* community. Previous findings obtained on several aquatic environments suggested that the dynamics of *Microcystis* genotypes are affected by factors specific to each environment because the conditions that allow for the proliferation, community composition formation, and mortality of *Microcystis* may involve various factors in the aquatic system. On a smaller scale as a comparison of two lakes, temperature and phosphate concentrations were identified as potential environmental factors affecting genotype dynamics, even though limnological parameters, including retention times and/or the stability of water, were also considered to affect genotype dynamics (Gagala *et al.*, 2014; Li *et al.*, 2014a). On the other hand, in a larger-scale study, in which a large number of aquatic systems were compared along strong environmental gradients (Marmen *et al.*, 2016) and/or among various environments within an aquatic system, such as Lake Erie (Rinta-Kanto *et al.*, 2009) and Lake Taihu (Li *et al.*, 2014a), some key factors and *Microcystis* genotypes were suggested. When attempting to identify key factors affecting microbial compositions, a large sample size covering an environmental gradient is needed (Langenheder and Ragnarsson, 2007; Marmen *et al.*, 2016).

To examine genotype distributions, we compared the *Microcystis* genotype composition among 88 aquatic systems across Japan, which is an island isolated from the continent. Three *Microcystis* genotypes, based on 16S–23S ITS sequence variations, and the gene copy number ratio of *mcyA* to IGS-PC were analyzed using qPCR. In addition, the relationship between *Microcystis* genotype compositions and environmental parameters was investigated to identify the key factors involved in the formation of *Microcystis* genotypic variations.

## Materials and Methods

### Sample collection and chemical analyses

**Fig. 1.**
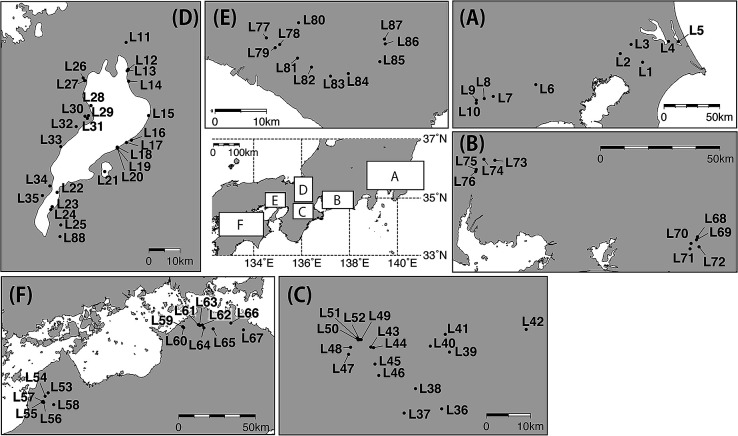
Location of 88 lacustrine sites that were sampled for *Microcystis* between October 9th and 31st, 2011.

Sampling was conducted in 88 freshwater environments, including ponds, reservoirs, dams, and lakes, across a 750-km area of Japan, between October 9th and 31st, 2011 (Fig. 1). Before sampling, *in situ* water temperature, conductivity, pH, and turbidity were measured at the shoreline (Hodoki *et al.*, 2013). Surface water samples were collected using a plastic bucket and stored in cold boxes for later analyses.

Two sets of 2–200-mL subsamples were filtered through pre-combusted (at 420°C for 3 h) GF/F filters (Whatman). The filters were wrapped in aluminum foil and stored at –20°C until genomic DNA extraction and chlorophyll *a* (Chl-*a*) concentration measurements. The filtrates were also stored at –20°C and used to analyze nutrients in the dissolved fraction. Nitrate (NO_3_), nitrite (NO_2_), and phosphate concentrations were examined colorimetrically using an AACS II Auto Analyzer (Bran+Luebbe, SPX), and total nitrogen (TN) and total phosphorus (TP) concentrations (including particle and dissolved fractions) were also measured colorimetrically after oxidation of the water sample with potassium persulfate (Pujo-pay and Raimbault, 1994). The ammonium concentration was measured fluorometrically (Holmes *et al.*, 1999). Total inorganic carbon (TOC) concentrations were measured using a Shimadzu TOC-5000 analyzer (Shimadzu). Chl-*a* was extracted with 10 mL of *N,N*-dimethylformamide (Suzuki and Ishimaru, 1990), and its concentration was measured using a spectrofluorophotometer (RF-5300. Shimadzu) following the method of Welschmeyer (1994).

### DNA extraction

Genomic DNA was extracted from the whole GF/F filter using potassium xanthogenate-sodium dodecyl sulfate with a variation of the phenol/chloroform/isoamyl-alcohol (PCI) procedure (Tillett and Neilan, 2000) and homogenizing with pre-combusted glass beads (diameter <106 μm) (Kataoka *et al.*, 2013).

### qPCR of the gene fragment of IGS-PC, *mcyA*, and intra-specific genotypes within the 16S–23S ITS sequence

Three major genotype variations, defined by sequence variations within the 16S–23S ITS region, were selectively detected by real-time quantitative PCR (qPCR) using the three group-specific PCR primer sets, listed in Table S1: G3f/MITS-R for *M. wesenbergii*-like non-toxic group (MG3), MITS-F/G4r for *M. viridis*-like toxic group (MG4), and MITS-F/G1r for *M. aeruginosa*-like group (MG1) organisms, including toxic and non-toxic *Microcystis* strains. qPCR reactions for the three groups were performed as described by Kataoka *et al.* (2013), and qPCR for the IGS-PC and *mcyA* genes according to the procedures described by Yoshida *et al.* (2007).

### Statistical analysis

Variance in environmental variables and *Microcystis*-related variables, such as IGS-PC, which is related to *Microcystis* abundance, and the ratio of *mcyA* to IGS-PC (*mcyA*/PC), which is an index of the toxic genotype ratio of *Microcystis*, were visualized using the qqnorm function in the stats package (ver. 3.3.2) for software R. Variables showing a non-normal distribution were transformed by the square or fourth root or log10 conversion. The relative value, the difference from the minimum value, was applied to longitude and temperature; the square root to conductivity and TOC; the fourth root to turbidity, Chl-*a*, TN, TP, dissolved inorganic nitrogen (DIN), and NO_3_; and the logarithmic transformation to dissolved inorganic phosphorus (DIP), NO_2_, NH_4_, and PC. Multicollinearity among variables was then assessed by variance inflation factors (VIF) to remove variables showing VIF>20 in order for data on DIN and DIP/DIN to be eliminated from subsequent analyses because of their strong correlation with NO_3_ concentrations. Latitude was also eliminated for its strong correlation with longitude. The detection of *Microcystis* in a sample was performed based on the threshold cycle (Ct) value of IGS-PC-based qPCR to remove samples showing Ct>30. Outliers in qPCR data were identified by referring to a clustering analysis and ordination plot of non-metric multidimensional scaling (nMDS). In the remaining samples, the relative abundance of three major genotypes of the *Microcystis*-specific 16S–23S ITS (MITS) region of MG1, MG3, and MG4, was calculated. After conducting pairwise comparisons of dissimilarity based on the Bray–Curtis distance, significant clusters among sampling sites were tested by a similarity profile analysis (SIMPROF: clustering package) with *P*<0.05. The dissimilarity of the genotype composition among sampling sites was visualized, and the effects of environmental factors on the genotype composition were tested by a redundancy analysis (RDA: vegan package). To test the significance of constraints among the constructed RDA models, a non-parametric multivariate ANOVA test was conducted. The effects of environmental variables on the dissimilarity of the community composition were tested by a permutational multivariate analysis of variance (PERMANOVA: an adonis2 package), and the contribution of each environmental variable was estimated by alternatively eliminating each environmental variable (partial RDA). All statistical analyses were conducted using R software ver. 3.3.2 (http://cran.r-project.org).

## Results

### Identification of outliers

Water from a pond at site L81 showed extremely eutrophic conditions with 5,344 μg L^–1^ of Chl-*a*, 206 μg L^–1^ of TOC, 77,390 μg L^–1^ of TN, and 7,130 μg L^–1^ of TP, and the highest gene copy number for IGS-PC of 7.2×10^7^ copies mL^–1^ and *mcyA* of 1.0×10^7^ copies mL^–1^, indicating a massive bloom of *M. aeruginosa*. Preliminary composition analyses, such as nMDS and cluster analyses, for the MITS genotypes (MG1, MG3, and MG4) showed that the *Microcystis* composition of a dam at site L36 markedly differed from that found in other sites. The MITS genotype composition of L36 was dominated by the MG4 genotype: 29.3% of the total MITS genotypes. Thus, sites L36 and L81 were excluded as outliers, which draw incorrect results.

### Environmental variables

Seventy out of the 88 sampling sites shown in Fig. 1 showed the presence of *Microcystis* populations, which were widely distributed throughout Japan. Sixty-eight sites, after the removal of the two outliers described above, showed significantly higher water temperatures (ranging between 13.8 and 22.6°C), pH values (ranging between 6.44 and 10.35), and TOC concentrations (ranging between 0.86 and 23 μg L^–1^) than the other 18 sites, at which no *Microcystis* populations were detected by IGS-PC-targeted qPCR (the Wilcoxon rank test: *P*<0.01) (Fig. S1). Chl-*a* concentrations ranged between 3.06 and 864 μgL^–1^ and correlated with both TN (Pearson’s correlation coefficient *R*^2^=0.76, *P*<0.01) and TP (Pearson’s correlation coefficient *R*^2^=0.77, *P*<0.01).

### *Microcystis* population and toxic genotype

The gene copy number of IGS-PC ranged between 1.2×10^2^ and 3.7×10^7^ copies mL^–1^ with an average of 9.4×10^5^ copies mL^–1^ (±4.5×10^6^ SD) (Fig. S1). The distribution of the IGS-PC gene copy number showed that 47 (69.1%) sites contained less than 1.0×10^5^ IGS-PC copies mL^–1^, whereas 12 (17.6%) and 9 (13.2%) sites showed a range of 1.0×10^5^ to 1.0×10^6^ copies mL^–1^ and more than 1.0×10^6^ copies mL^–1^, respectively, with a median of 1.1×10^4^ copies mL^–1^ (Fig. S2A). The gene copy number of *mcyA*, which ranged between 1.4×10^1^ and 3.6×10^6^ copies mL^–1^ (1.1×10^5^ copies mL^–1^ on average, ±4.6×10^5^ SD), strongly correlated with the IGS-PC copy number (*R*^2^=0.91) (Fig. S2B). The percentage index of *mcyA*/PC was the highest (50.01%) at one site. Twenty-six sites (38.2%) were in the range of <10%, followed by 19, 12, 9, and 1 sites in the range of 10–20, 20–30, 30–40, and 40–50%, respectively, with a median of 16.3%.

### Correlation analysis of IGS-PC and *mcyA*/PC to environmental variables

The pairwise correlation analysis revealed a correlation between the gene copy number of both IGS-PC and *mcyA*/PC with environmental variables. The IGS-PC gene correlated with TN (*r*=0.55), TP (*r*=0.55), and TOC (*r*=0.66), and IGS-PC strongly correlated with turbulence (*r*=0.69) and Chl-*a* (*r*=0.75) (*P*<0.05). However, *mcyA*/PC only showed a weak correlation with some variables (*r*<0.4) (Table 1). The correlation patterns of IGS-PC were similar at sampling sites with a high IGS-PC gene copy number, >1.0×10^5^ copies mL^–1^ (21 sites), whereas no correlation was observed in samples with a lower IGS-PC gene copy number, <1.0×10^5^ copies mL^–1^ (47 sites) (Table S2).

### Composition of 16S–23S ITS genotypes and efficient environmental factors

**Fig. 2.**
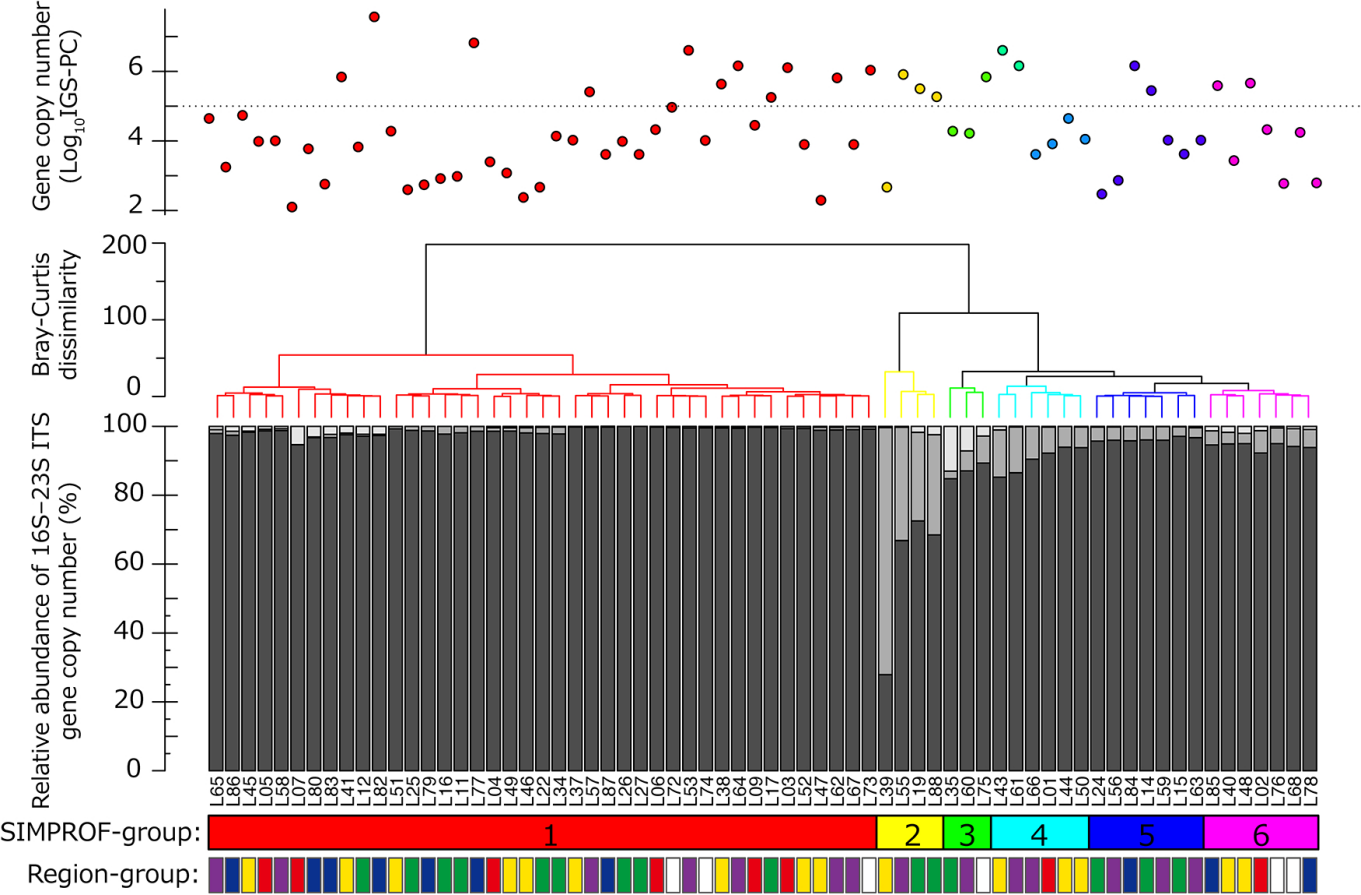
*Microcystis* genotypic composition described by the relative abundance of three genotypes, MG1, MG3, and MG4 (bottom). The composition of each site is grouped according to the SIMPROF analysis based on the Bray–Curtis distance (*P*<0.05) (middle). The abundance of the IGS-PC gene copy number is shown (top). Dark gray, pale gray, and white in the bottom panel indicate the *Microcystis* genotypes of MG1, MG3, and MG4, respectively. The color charts below the bottom panel show the groups as a result of the SIMPROF analysis (S-group) and the geographical groups as described in Fig. 1. Red, white, yellow, green, blue, and purple correspond to the regions A, B, C, D, E, and F, respectively, in Fig. 1. The dotted line in the top panel shows the threshold IGS-PC gene copy abundance between high and low abundance communities (10^5^ copies mL^–1^).

SIMPROF showed that the composition of *Microcystis* genotypes derived from 16S–23S ITS, *i.e.*, MG1, MG3, and MG4, may be separated into five significant groups (SIMPROF, *P*<0.05; Fig. 2). In the largest SIMPROF group (41 sites), 94.7% of the MITS genotypes were MG1 and less than 2.3 and 5.3% were MG3 and MG4, respectively. Of the other distant SIMPROF-groups, S-group-2 was dominated by MG3, representing 25.7–71.7%, and MG4, representing less than 2.4% of the MITS genotypes; S-group-3 was dominated by MG4, occupying 2.8–13.0%, and MG3, representing 2.2–7.9% of the MITS genotypes. The remaining three SIMPROF-groups, S-group-4 to -6, showed similar genotype patterns to each other, with MG1 being dominant, followed by MG3 and MG4.

**Fig. 3.**
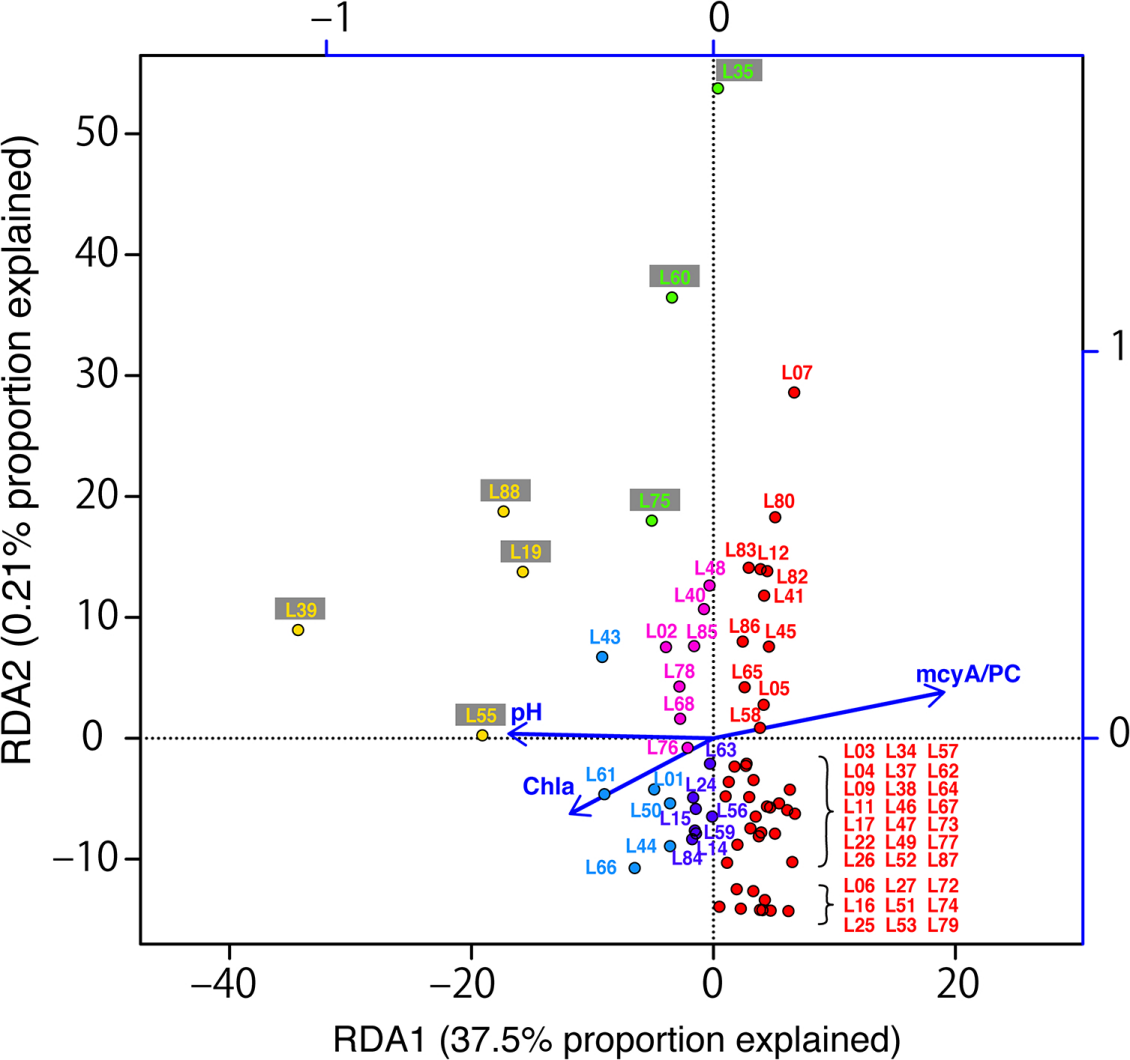
RDA analysis based on *Microcystis* genotypic composition. Color corresponds to the grouping as a result of the SIMPROF analysis shown in Fig. 2. Chl-*a* and *mcyA*/PC indicate chlorophyll *a* concentrations and the ratio of *mcyA* to the IGS-PC gene copy number, respectively. The length and direction of the arrows indicate the contribution of environmental variables.

The six groups also emerged in RDA, in which 40.0% of the total community variation was explained by environmental variables *P*<0.05) (Fig. 3). Of the total variation in the MITS community composition, 37.5% was explained along the RDA1 axis, whereas only 0.21% was along the RDA2 axis. S-group-1, S-group-2, and S-group-3 were distantly located along the RDA1 axis, and even S-group-3 was scattered along the RDA2 axis. In contrast, the other SIMPROF-groups, S-group-4 to -6, were dispersed less in the RDA plot. pH, Chl-*a*, and *mcyA*/PC significantly affected the MITS genotype composition (PERMANOVA, *P*<0.05). Partial RDA showed that *mcyA*/PC and pH strongly contributed to total variance, representing 10.7 and 8.1% of the total variance, respectively. Of the significant environmental variables identified, pH was positively affected in S-group-2, which was dominated by the MITS genotype MG3, and *mcyA*/PC was positively affected in S-group-1, which was dominated by MG1. The correlation analysis also showed that pH positively correlated with MG3 (*r*=0.31, *P*<0.05) and negatively correlated with MG1 (*r*=–0.29, *P*<0.05) (Table 1). Chl-*a* concentrations, which slightly contributed to the MITS genotype composition (1.9% of total variance), were affected in S-group-4 to -6.

## Discussion

### Composition and abundance of 16S–23S ITS genotypes

The three genotype groups of the 16S–23S ITS region, corresponding to MG1, MG3, and MG4, detected by the full-length sequencing analysis of the 16S–23S ITS region, were dominant in *Microcystis* obtained from 12 lacustrine sites in western Japan (Ohbayashi *et al.*, 2013). This agreement between culture-dependent and culture-independent analyses supports the accurate detection of *Microcystis* genotypes in the present study. The dominance of MG1 was observed irrespective of the bloom scale, which was estimated by IGS-PC gene abundance (top panel in Fig. 2), but weakly correlated with *mcyA*/PC (Table 1). This suggests that the MG1 genotype is widely distributed across Japan, irrespective of the bloom phase because our samples may have included the early, middle, and late phases of *Microcystis* bloom. These results correspond to previous findings showing that the 16S–23S ITS genotypes were highly heterogeneous, and some were globally distributed (Janse *et al.*, 2003; 2004). Moreover, the primers MG1 were designed to detect various *Microcystis* phenotypes, including microcystin-producing and non-microcystin-producing strains, and several morphospecies, including *M. aeruginosa*, *M. flos-aquae*, and *M. ichthyoblabe*, which have been detected worldwide (Kataoka *et al.*, 2013). Although the genotype variation of *Microcystis*, assessed by the 16S–23S ITS region-based denaturing gradient gel electrophoresis analysis, was relatively low in several freshwater environments (Kardinaal *et al.*, 2007), a recent comparison of a large number of sites revealed that the genetic diversity of the 16S–23S ITS region was irrespective of geographic distance (Ohbayashi *et al.*, 2013). Temporal surveys have shown that the genotypic compositions of *Microcystis* markedly changed from before to after bloom formation (Yoshida *et al.*, 2005; Kardinaal *et al.*, 2007). The sites at which the MG1 genotype dominated require more intense investigations using temporal surveys and a focus on the toxic genotypes within MG1. Furthermore, the diversity within MG1 needs to be examined in more detail using a whole-genome analysis (Yamaguchi *et al.*, 2018).

MG3 was the second most dominant genotype and was also distributed across sampling sites, irrespective of IGS-PC gene abundance (Fig. 2). This genotype includes only a single morphospecies of *M. wesenbergii* (Kataoka *et al.*, 2013), which does not produce microcystins (Kurmayer *et al.*, 2002; Xu *et al.*, 2008). Of the 68 sampling sites at which the *Microcystis* IGS-PC gene was detected, 27 (40%) contained MG3, even at sites at which the relative abundance of *Microcystis* was less than 33% (L55). However, at the highest relative MG3 abundance of 72% (site L39), there was low IGS-PC gene abundance (approximately 4,000 IGS-PC gene copies mL^–1^). The corresponding morphospecies of *M. wesenbergii* are globally distributed, but are less frequently found at high concentrations than *M. aeruginosa*. In other words, cyanobacteria blooms are less frequently dominated by *M. wesenbergii* in Europe (Via-Ordorika *et al.*, 2004). However, this morphospecies has been reported to regularly form massive blooms in aquatic ecosystems in China (Xu *et al.*, 2008). Imai *et al.* (2009) reported that morphospecies of *M. wesenbergii* co-existed with *M. aeruginosa* in Japan and occupied between 10 and 80% of the phytoplankton composition throughout the year in a dam lake. The distribution pattern of the genotype MG3 strongly agrees with the distribution pattern of *M. wesenbergii* detected by both molecular and microscopy analyses. Thus, the present results indicate that the distribution of the MG3 genotype is not limited by distance, but is rarely a highly abundant member of the phytoplankton community in Japan. However, based on biomass, the large cell volume and thick mucilaginous sheath of *M. wesenbergii* indicate that these phytoplankton (Komárek, 1958; Imai *et al.*, 2009) are sufficiently common to be very problematic.

The genotype MG4, which exclusively included microcystin-producing toxic morphotypes of *M. viridis*, rarely dominated sites in the present study, indicating their limited distribution or that they are limited by the overwhelming abundance of the other genotypes of *Microcystis* or other phytoplankton. The absence of any correlation between MG4 abundance and *mcyA*/PC (Table 1) may be attributed to insufficient sampling efforts. To expand our knowledge on how the *Microcystis* community composition is formed, further studies on environmental factors and/or competition mechanisms among these genotypes are needed.

### Toxic genotype abundance and environmental factors

The pairwise correlation analysis was used to examine relationships between environmental factors and *Microcystis* abundance and between environmental factors and *mcyA*/PC (Table 1 and S2). *Microcystis* abundance, estimated by the IGS-PC gene copy number, positively correlated with TN and TP (*P*<0.05) when total samples (68 samples) and high-IGS-PC samples (21 sites: 1.0×10^5^ copies mL^–1^ >PC) were both analyzed. Non-nitrogen-fixing *Microcystis* depend on inorganic nutrient availability (Orr and Jones, 1998; Gobler *et al.*, 2016). On the other hand, low-nutrient conditions, which were defined as concentrations of less than 400‍ ‍μg‍ ‍L‍^–1^ for TN and/or 20 μg L^–1^ for TP (Fujimoto *et al.*, 1997), were observed at 18 sites, and these sites also showed low *Microcystis* abundance (2.0×10^2^ to 1.8×10^4^ copies mL^–1^ of the IGS-PC gene) or levels below the detection limit. Although TN and/or TP have been shown to correlate with toxic cyanobacteria abundance (Vézie *et al.*, 2002; Davis *et al.*, 2009; Li *et al.*, 2014b), it is important to note that *Microcystis* itself contributes to TN, TP, and TOC concentrations. Although the present results showed no correlation between the index of *mcyA*/PC and environmental factors, the gene abundance of *mcyA* and IGS-PC positively correlated in a log–log scale comparison (*R*^2^=0.91, Fig. S2B). Regarding other environmental factors, genotype composition was affected by pH, and the proportion of the MG3 genotype and pH positively correlated (Table 1 and Fig. 3). To date, culture studies have shown that *Microcystis* preferentially grow in neutral to weak alkaline conditions, *i.e.*, pH>6.5 (Wang *et al.*, 2011). Of our 88 samples, 68 sampling sites at which the IGS-PC gene was detected had a pH>7.0, excluding site L27 at which we detected a pH of 6.44 and low IGS-PC gene abundance. Therefore, a neutral to alkaline pH environment appears to be a significant factor for the maintenance of *Microcystis* populations. More specifically, Yang *et al.* (2018) showed that a pH in the range of 7–8 was optimum for *M. aeruginosa* strain 7806, which belongs to MG1 in our genotype grouping, and conditions under pH 6 and over pH 9 were hostile to growth. The present results also showed that pH 7–8 was optimum for *Microcystis* populations because the majority of MG1-dominated populations (65.9%, 27/41), which were designated as S-group-1, were derived from sites with a pH in this range. In addition, IGS-PC was detected in only four sites at which pH was >9, with the maximum pH value being 9.68. However, for MG3-abundant populations, designated as S-group-2 and S-group-4 to -6, the IGS-PC gene was detected up to pH 10.4. Since pH increases as *Microcystis* blooms grow because of CO_2_ consumption by photosynthesis (Paerl and Ustach, 1982; Krausfeldt *et al.*, 2019), dominant morphotypes in the late bloom phase may be adapted to high pH. *M. aeruginosa* and *M. wesenbergii* formed large colonies in the late bloom phase between August and October in Lake Taihu (Li *et al.*, 2016; Xiao *et al.*, 2018). Thus, MG3 may survive at the late bloom phase. The predominance of *Microcystis* under high pH conditions has been reported in the natural phytoplankton community (Shapiro, 1997) and in experimental co-cultures, including *Microcystis* and a chlorophyta (Nakano *et al.*, 2003). Shapiro (1997) showed that *Microcystis* exhibited relatively low photosynthesis activity under high pH conditions, suggesting that under these conditions, *Microcystis* enters a state of maintenance with low levels of reproduction. This tolerance to a high pH may be why the species dominates over other phytoplankton (Nakano *et al.*, 2003). The present results indicate that pH is an important factor for community compositional differences at the intra-species genotype level. Moreover, pH positively correlated with MG3 and negatively correlated with MG1 relative abundance, supporting a previous study indicating a negative correlation between *mcyD* abundance and pH (Rinta-Kanto *et al.*, 2009). pH also appears to be one of the driving forces behind the formation of harmful and/or non-harmful *Microcystis* blooms.

### Methodological considerations

This wide area, short-term survey of *Microcystis* populations revealed that one genotype, MG1, dominated the most in lacustrine environments in Japan. We also succeeded in identifying some of the environmental factors affecting *Microcystis* genotype composition, *e.g.*, pH, and those affecting *Microcystis* abundance, *e.g.*, TN and TP. However, it is important to note that in our snapshot survey, difficulties may be associated with completely elucidating *Microcystis* population dynamics because cyanobacteria blooms are seasonal events, and cyanobacteria populations respond to temporary environmental variations (Yoshida *et al.*, 2007; Rinta-Kanto *et al.*, 2009). For example, Yoshida *et al.* (2007) found a positive correlation between the *mcyA*/PC ratio and NO_3_, whereas no correlation was found in the present study. Furthermore, the composition of the three genotypes defined by the 16S–23S ITS region did not reveal any relationships between genotype compositions and aquatic environment categories (Table S3; *e.g.*, dam, pond, lake, and marsh) or geographic distances (Fig. S3) because we analyzed genotype abundance rather than genotype composition. Temporal and wide area surveys targeting genotypic diversity (*e.g.*, metagenome analyses and/or whole-genome types) are both needed to obtain further insights into *Microcystis* population dynamics.

## Supplementary Material

Supplementary Material

## Figures and Tables

**Table 1.** Correlation coefficients between environmental factors, ITS genotype relative abundance, *Microcystis* IGS-PC gene abundance, and the *mcyA*/PC ratio.

	Lon	Tem	pH	Cond	Turb	Chl-*a*	TOC	TN	TP	DIP	NO3	NO2	NH4	IGS-PC	mcyA/PC	MG1	MG3	MG4
Lon	1																	
Tem	–0.27**	1																
pH	–0.16	0.36***	1															
Cond	0.31**	–0.25**	0.01	1														
Turb	–0.10	0.02	0.11	0.22	1													
Chl-*a*	0.00	–0.02	0.27**	0.33**	0.83***	1												
TOC	–0.18	0.12	0.28**	0.23	0.77***	0.82***	1											
TN	–0.06	–0.01	0.13	0.29**	0.76***	0.75***	0.7***	1										
TP	–0.01	0.02	0.07	0.29**	0.77***	0.78***	0.79***	0.78***	1									
DIP	0.06	–0.02	–0.17	0.16	0.17	0.09	0.17	0.3**	0.57***	1								
NO3	0.21	–0.26	–0.33**	0.17	–0.28	–0.35***	–0.45***	0.12	–0.19	0.27**	1							
NO2	0.28**	–0.35	–0.31**	0.32**	–0.09	–0.19	–0.33**	0.17	–0.04	0.34***	0.87***	1						
NH4	0.10	–0.36	–0.34**	0.18	–0.05	–0.13	–0.21	0.09	0.00	0.19	0.5***	0.62***	1					
IGS-PC	–0.22	0.23	0.34***	0.12	0.69***	0.75***	0.66***	0.55***	0.55***	–0.01	–0.4***	–0.29**	–0.12	1				
mcyA/PC	–0.19	–0.02	–0.09	–0.41***	–0.18	–0.4***	–0.24	–0.27**	–0.31**	–0.07	0.04	0.07	0.06	–0.3**	1			
MG1	0.07	–0.02	–0.29**	0.00	0.09	–0.10	–0.08	–0.08	–0.02	0.04	–0.04	0.02	0.03	0.03	0.32**	1		
MG3	–0.07	0.02	0.31**	0.01	–0.06	0.13	0.09	0.12	0.04	–0.06	0.05	–0.01	–0.04	–0.03	–0.33**	–0.98***	1	
MG4	0.00	–0.03	–0.07	–0.08	–0.14	–0.11	–0.08	–0.18	–0.10	0.09	–0.07	–0.06	0.05	–0.03	0.02	–0.19	0.00	1

** *P*<0.05; *** *P*<0.01
