# Supplementary material for: Distribution of the Harmful Bloom-Forming Cyanobacterium, Microcystis aeruginosa, in 88 Freshwater Environments across Japan
Source: Microbes Environ. 2020 Feb 20;35(1):ME19110. doi: 10.1264/jsme2.ME19110 (PMC7104289; doi:10.1264/jsme2.ME19110)
Supplement: Supplementary file 1 — Supplementary Material [file 35_19110_s1.pdf]

Table S1. Positions, characteristics, and sequences of the primers used for intra-specific selective detection of *Microcystis* genotypes.

Primer name	Position in ITS region	Target genotype	F/R	Sequences	Reference
G1r	46–65	*Microcystis aeruginosa*-like	R	CGGAG CGAGG CGAAA TT	Kataoka et al., 2013
G3f	7–26	*Microcystis wesenbergii*-like	F	GACGA AAAAA TAGTA GTCGA AA	Kataoka et al., 2013
G4r	235–256	*Microcystis viridis*-like	R	CAGCA CCTTA TCTTA TATAC ATATA	Kataoka et al., 2013
MITS-F	–	–	F	AAGGG AGACC TAATT CVGGT	Yoshida et al., 2008
MITS-R	–	–	R	TTGCG GTCYT CTTTT TTGGC	Yoshida et al., 2008

Table S2. Correlation coefficients between environmental factors versus Microcystis IGS-PC or *mcyA* gene abundance in samples where abundance of IGS-PC was (A) high (>10^5 copies mL^{-1}, n=21) and (B) low (<10^5 copies mL^{-1}, n=47).

	Lon	Tem	pH	Cond	Turb	Chl-*a*	TOC	TN	TP	DIP	NO3	NO2	NH4	IGS-PC	mcyA/ PC	MG1	MG3	MG4
Abundant samples (IGS-PC gene copy > 10^5 copies/mL, n=21)																		
**Lon**	1																	
**Tem**	0.33	1																
**pH**	0.12	0.47**	1															
**Cond**	-0.49**	-0.40	0.13	1														
**Turb**	0.23	-0.13	-0.22	0.10	1													
**Chl-*a***	0.07	-0.11	-0.09	0.36	0.83***	1												
**TOC**	0.00	0.25	0.19	0.05	0.57**	0.70***	1											
**TN**	0.01	0.02	-0.12	0.25	0.80***	0.80***	0.64***	1										
**TP**	0.25	0.14	0.01	0.19	0.73***	0.83***	0.65***	0.75***	1									
**DIP**	0.15	0.13	0.18	0.13	0.07	0.19	0.19	0.01	0.56**	1								
**NO3**	-0.30	-0.16	-0.17	0.31	-0.16	-0.14	-0.43	0.07	-0.17	-0.10	1							
**NO2**	-0.13	-0.28	-0.21	0.26	-0.01	-0.12	-0.45**	0.07	-0.08	0.09	0.91***	1						
**NH4**	-0.11	-0.59**	-0.44	0.00	0.16	-0.03	-0.33	-0.05	-0.10	-0.05	0.33	0.48**	1					
**IGS-PC**	0.25	0.15	-0.17	0.13	0.82***	0.79***	0.51**	0.74***	0.70***	0.02	-0.11	-0.09	-0.19	1				
**mcyA/PC**	0.10	0.20	0.00	-0.44	0.01	-0.21	-0.08	0.06	0.14	0.37	0.15	0.28	0.13	-0.06	1			
**MG1**	0.47**	-0.03	-0.41	-0.30	0.12	-0.05	-0.26	0.21	0.10	-0.04	0.12	0.16	0.09	0.16	0.43	1		
**MG3**	-0.44	0.07	0.46**	0.31	-0.11	0.02	0.21	-0.18	-0.12	-0.07	-0.09	-0.15	-0.09	-0.13	-0.46**	-0.98***	1	
**MG4**	-0.16	-0.21	-0.17	0.00	-0.06	0.12	0.25	-0.12	0.12	0.53	-0.17	-0.08	-0.01	-0.14	0.12	-0.17	-0.05	1
Not abundant samples (IGS-PC gene copy < 10^5 copies/mL, n=47)																		
**Lon**	1																	
**Tem**	-0.41***	1																
**pH**	-0.07	0.26	1															
**Cond**	0.49***	-0.22	-0.06	1														
**Turb**	0.2	-0.07	-0.31**	0.35**	1													
**Chl-*a***	0.44***	-0.16	-0.01	0.46***	0.58***	1												
**TOC**	0.08	-0.06	-0.13	0.34**	0.62***	0.65***	1											
**TN**	0.22	-0.16	-0.13	0.36**	0.52***	0.52***	0.50***	1										
**TP**	0.21	-0.15	-0.36**	0.37**	0.64***	0.60***	0.72***	0.66***	1									
**DIP**	0.06	-0.1	-0.48***	0.17	0.29**	0.03	0.19	0.52***	0.7***	1								
**NO3**	0.18	-0.26	-0.23	0.18	0.03	-0.13	-0.25	0.57***	0.07	0.47***	1							
**NO2**	0.30**	-0.36**	-0.25	0.37**	0.2	0.04	-0.1	0.54***	0.2	0.49***	0.86***	1						
**NH4**	0.09	-0.28	-0.29	0.22	0.01	-0.07	-0.11	0.29	0.14	0.28	0.52***	0.66***	1					
**IGS-PC**	0.11	0.26	0	0.18	0.09	0.30**	0.18	0.02	0.06	-0.12	-0.19	-0.17	0.01	1				
**mcyA/PC**	-0.34**	-0.06	-0.02	-0.41***	-0.13	-0.45**	-0.2	-0.33**	-0.4**	-0.22	-0.07	-0.05	0.02	-0.34**	1			
**MG1**	0.01	-0.01	-0.33**	0.06	0.11	-0.19	-0.07	-0.22	-0.07	0.07	-0.07	-0.01	0.02	0.01	0.31**	1		
**MG3**	-0.02	0.01	0.34**	-0.04	-0.08	0.23	0.1	0.26	0.09	-0.06	0.08	0.02	-0.03	-0.03	-0.31**	-0.98	1	
**MG4**	0.01	0.04	0.02	-0.1	-0.18	-0.17	-0.16	-0.2	-0.14	-0.06	-0.08	-0.08	0.06	0.09	-0.02	-0.19	0.01	1

**** p<0.05; *** p<0.01**

Table S3. *P*-values and *R*-values for grouping tested via analysis of similarity (ANOSIM), which test the significant difference between grouping. Aquatic categories A and B shows test for aquatic environment categories: A tested dam, lake, pond, marsh, B tested dam, lake, pond (pond and marsh in A). Geographic categories C–E shows test for regions in diferent scales: KNT, TOK, KS2, BWK, KS2, and SKK corresponds to Fig. 1A, 1B, 1C, 1D, 1E and 1F, respectively. KNT1 and 2 and SKK1 and 2 indicate the western and eastern part of the KNT and SKK, respectively.

	Categories	*R*-value*	*P*-value
Aquatic category			
A	Dam, Lake, Pond, Marsh	0.021	0.334
B	Dam, Lake, Pond	0.03	0.312
Geographic categroy			
C	KNT, KS, SKK, TOK	-0.025	0.633
D	KNT, KS1, KS2, BWK, SKK, TOK	-0.038	0.895
E	KNT1, KNT2, KS1, KS2, BWK, SKK1, SKK2, TOK	-0.029	0.725

* *R*-value was calculated based on the difference of mean ranks between groups and within groups, and ranges between -1 and 1. The value 0 indicates completely random grouping.

** The statistical significance of the *R*-value is assessed by 999 times permuting community composition to obtain the empirical distribution of *R*-value under null-model.

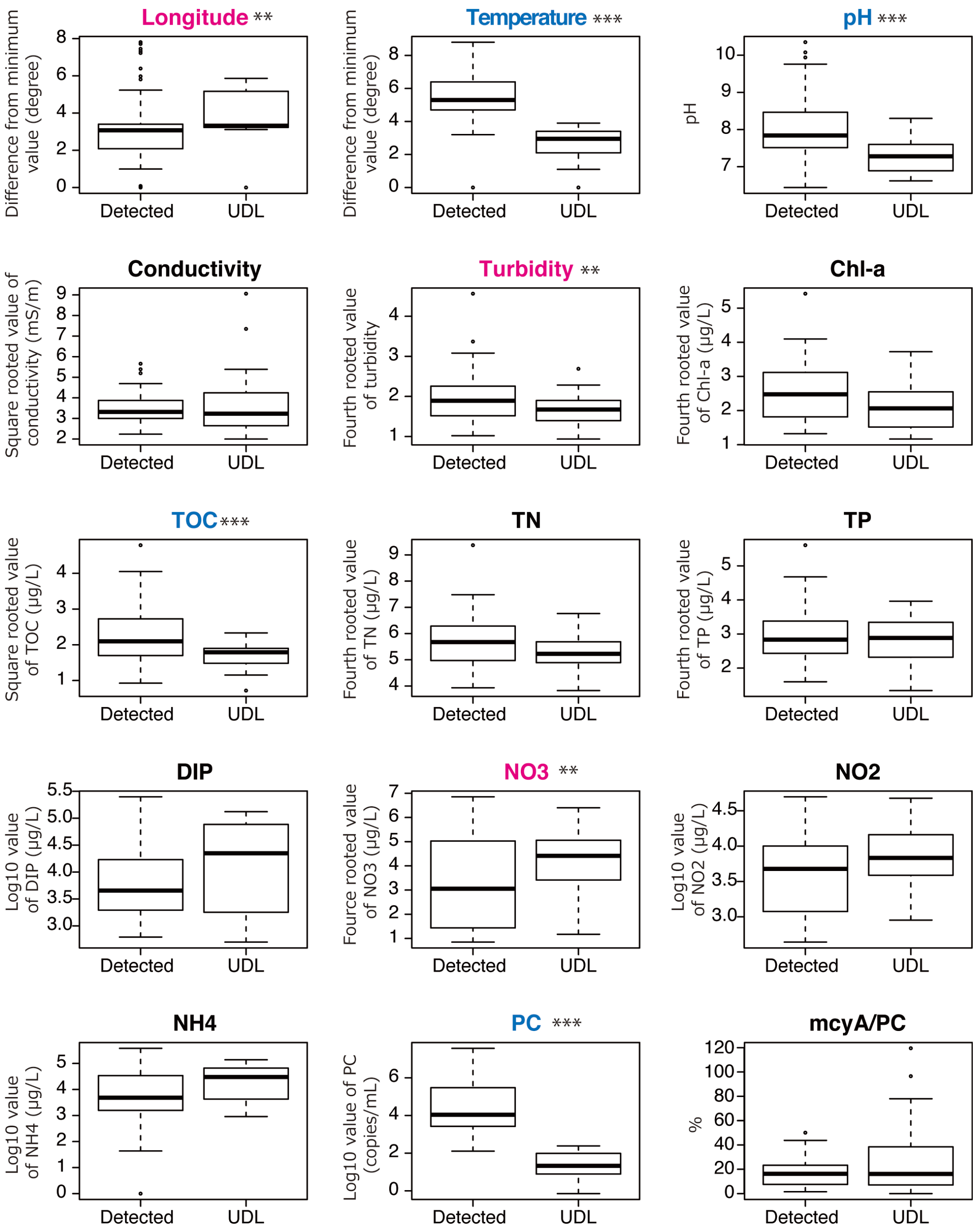

Fig. S1. Distribution of each variable in the present study. The labels Detected and UDL indicate above and under the detection limit of *Microcystis* IGS-PC gene copies based on the Ct value of 30, respectively. Samples of the Detected and UDL include 68 and 18 samples, respectively. Two samples were excluded as outliers. The analyzed variables were converted to an approximately normal distribution. Relative value, the difference from the minimum value, was applied to longitude and temperature; square root was applied to conductivity and TOC; fourth root was applied to turbidity, Chl-*a*, TN, TP, DIN, and NO3; logarithmic transformation was applied to DIP, NO2, NH4, and PC. Chl-*a*, TN, TP, DIN, NO3, NO2, and NH4 indicate chlorophyll *a* concentration, total nitrogen, total phosphate, dissolved inorganic nitrogen, nitrate ion, nitrite ion and ammonium ion, respectively. Asterisks indicate the *p*-values of Wilcoxon's rank test: *** and ** indicate $p<0.01$ and $p<0.05$, respectively.

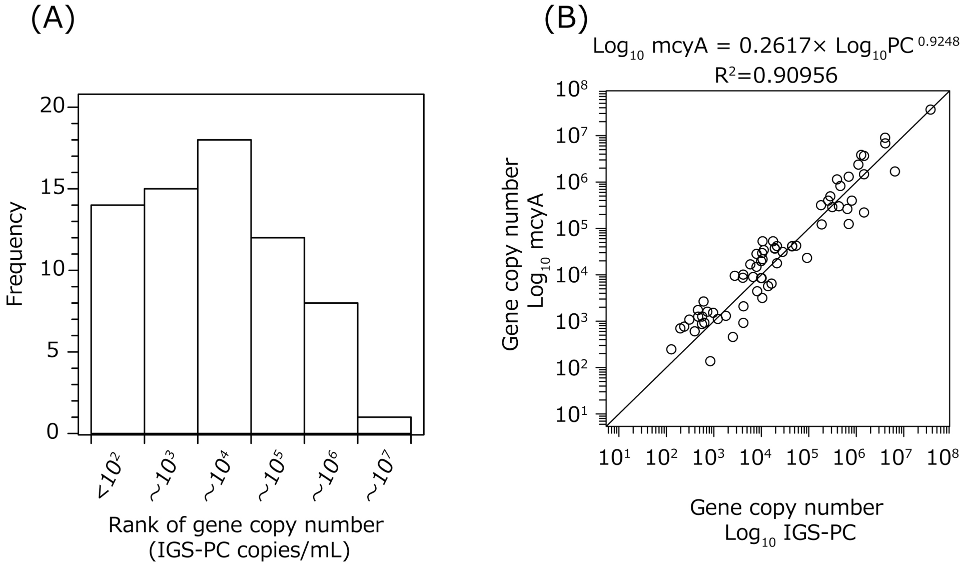

Fig. S2. Frequency of IGS-PC gene copy number in 68 samples (A) and relationship between *mcyA* and IGS-PC gene copy number (B).

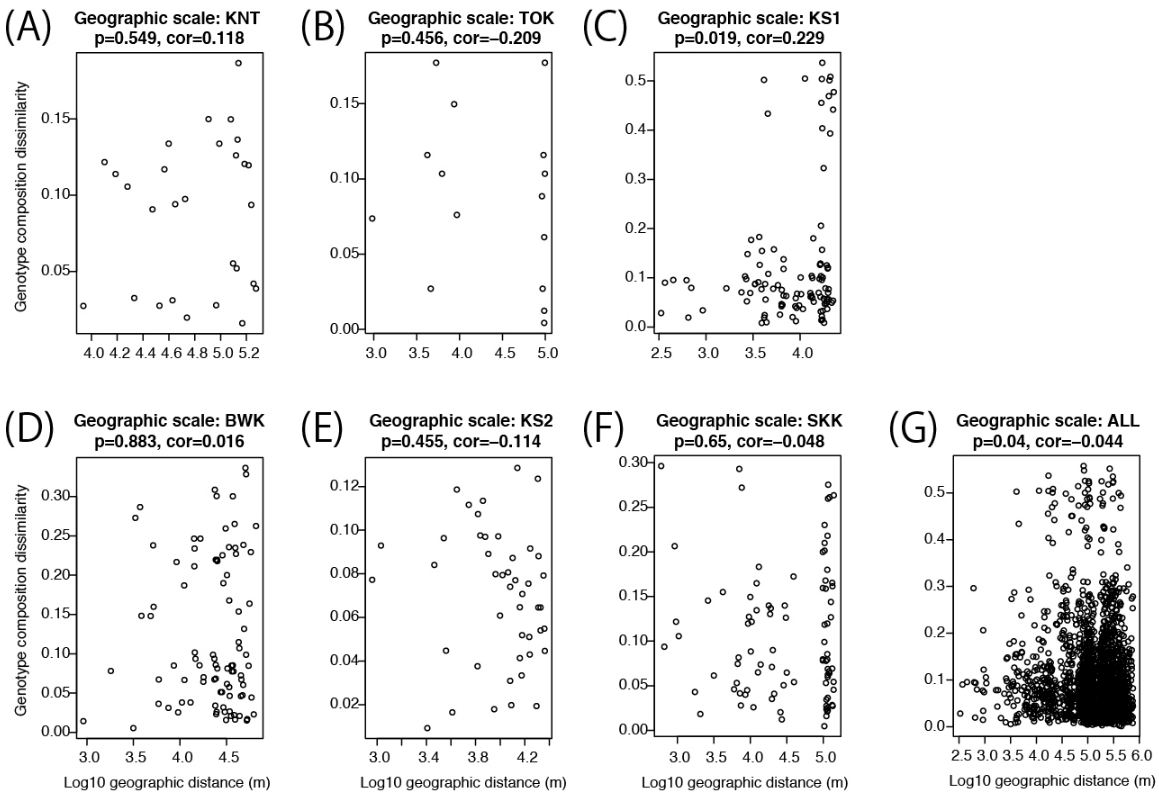

Fig. S3. Scatter plot of genotype composition dissimilarity, which was calculated using a Bray-Curtis distance from a pair of square-root converted genotypic compositions, and log10 converted geographic distance (m). Panel A–F correspond to region in the panel A–F in Fig. 1, respectively, and panel G includes all pairs. The geographic scale of KNT, TOK, KS2, BWK, KS2, and SKK corresponds to Fig. 1A, 1B, 1C, 1D, 1E and 1F, respectively. P-value and correlation coefficient (cor) of Pearson’s correlation test are shown on the top of panel.
